# Development of active tuberculosis during treatment of head and neck carcinoma: a case series

**DOI:** 10.1186/s13256-019-2055-2

**Published:** 2019-05-24

**Authors:** Mioko Matsuo

**Affiliations:** Department of Head and Neck Surgery, Japan Community Health Care Organization Kyushu Hospital, 1-8-1 Kishinoura, Yahatanishi-ku, Kitakyushu City, Fukuoka 806-8501 Japan

**Keywords:** Head and neck carcinoma, Active tuberculosis, Prophylactic administration

## Abstract

**Background:**

Patients with head and neck carcinoma are considered to be at high risk of developing tuberculosis, since the risk of morbidity due to tuberculosis in these patients is 2.86 to 16 times the risk in the general population.

**Case presentation:**

This case series describes an 83-year-old Japanese man, a 60-year-old Japanese man, and a 69-year-old Japanese man who developed active pulmonary tuberculosis while being treated for head and neck carcinoma. Two had previously developed tuberculosis and were treated for more than 50 years, but no symptoms or imaging findings suggested tuberculosis onset in the patients at initiation of treatment for head and neck carcinoma. Initially, local radiotherapy was performed for all three patients. Chemotherapy was continued for two patients who had pulmonary metastasis since initial consultation. For the other patient, surgery was performed for recurrence. In all three cases, active tuberculosis infection was observed during maintenance chemotherapy or immediately following surgery.

**Conclusions:**

Due to the high risk of developing tuberculosis, the possibility of prophylactic administration of anti-tuberculosis agents to patients with head and neck carcinoma should be investigated, although prophylactic administration is not a cost-effective option for all patients with head and neck carcinoma. However, if tuberculosis onset occurs, it leads to various problems; it has a major impact on not only patients with cancer but also various people in the social environment. In the future, it is essential to consider prophylactic administration in patients requiring long-term maintenance drug therapy, especially in those who are treated at out-patient chemotherapy clinics, where there are several patients with cancer with low disease resistance.

## Background

The global incidence of tuberculosis (TB) is declining every year [1]. However, even in 2017, TB was only second to human immunodeficiency virus (HIV) in the number of deaths caused [1]. Furthermore, even in countries with low TB incidence, there has recently been an increase in the number of patients in the high-risk group for TB, which includes those who have HIV infection, have undergone organ transplantation, or are undergoing hemodialysis because of chronic renal failure; therefore, there are new issues in relation to the measures for treating TB [2, 3]. In addition to the high-risk group for TB, patients with malignant neoplasms are considered to have an increased risk of developing TB; among the different types of malignancies, head and neck carcinoma is associated with the second highest risk group for TB after hematological malignancies [3–5]. However, there is no consensus on the approach to the treatment of head and neck carcinoma and/or TB in patients who develop active pulmonary TB during treatment for head and neck cancer [6, 7]; as a countermeasure, the treatment is stagnated.

I report the cases of three patients who developed active TB during treatment for head and neck carcinoma; they were selected from patients with head and neck cancer who received treatment during the 3 years from January 2015 to December 2017. The frequency at which active pulmonary TB develops during treatment of head and neck carcinoma is reported to be approximately 0.5% [5]; therefore, these three rare cases are believed to be useful for future reference.

## Case presentation

These reports were approved by the Community Health Care Organization Kyushu Hospital, Japan.

### Case 1

An 83-year-old Japanese man was diagnosed as having laryngeal squamous cell carcinoma (T2N0M0) at the Department of Head and Neck Surgery, Kyushu Hospital, in November 2016. He had been treated for pulmonary TB at the age of 18, but on examination, thoracic computed tomography (CT) and positron emission tomography (PET) showed no thoracic abnormalities. Radiotherapy for laryngeal cancer at 70 Gy (35 fr) was performed and the tumor disappeared. However, in April 2017, primary lesion recurrence with laryngeal edema and cervical lymph node metastasis were observed. While waiting for surgery, steroid (prednisolone) was administered for 1 month, with the dose starting at 60 mg and being reduced gradually, with the aim of alleviating the edema. In May 2017, a total laryngectomy and bilateral cervical lymph node dissection were conducted.

No thoracic abnormalities were observed on initial examination in November 2016, but a thoracic X-ray 2 weeks before surgery in May 2017 revealed a small amount of pleural effusion. The amount of pleural effusion increased immediately after surgery, and by the following day he had developed fever (39 °C). At first, the condition was considered to be pleural effusion associated with pneumonia due to general bacteria, and sulbactam/ampicillin and meropenem were administered, but alleviation of symptoms was not achieved. Pleural fluid analysis showed that lymphocytes were present, abating the concern for bacterial infection. The possibility of TB was considered; therefore, sputum smear tests, including rapid molecular diagnostic testing for TB using real-time polymerase chain reaction (PCR), were conducted five times, but the results were negative. *Mycobacterium tuberculosis* was detected in a solid medium (“Ogawa” medium) culture test after 3 weeks; our patient was considered to have TB pleural effusion, and TB treatment was initiated. He continued to receive anti-TB drugs, which were rifampicin (RFP), isoniazid (INH), and ethambutol (EB), but died 2 months later because his general condition subsequently deteriorated. Because he had a history of TB infection, the possibility of TB was considered unlikely; therefore, an interferon-γ release assay (IGRA) test was not performed.

### Case 2

**Fig. 1:**
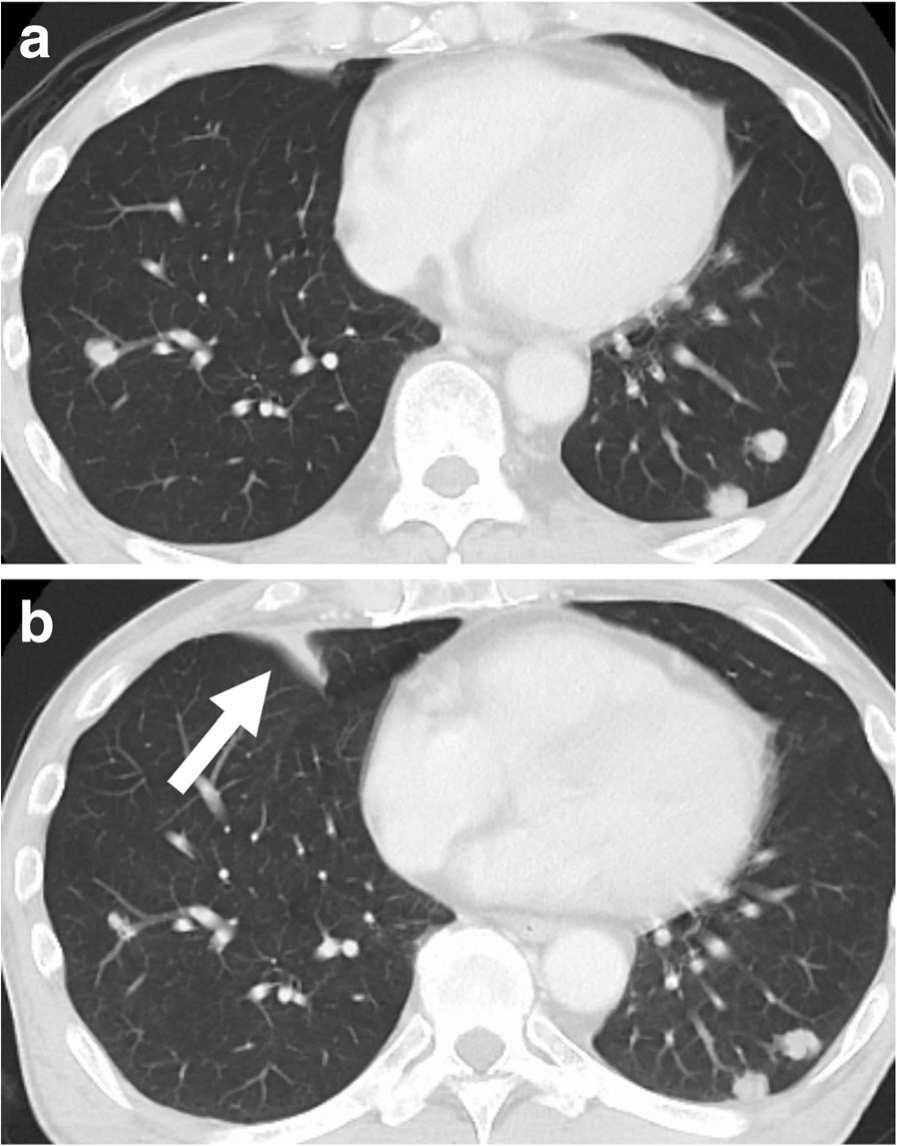
Chronological changes in the lungs for Case 1 as shown usingcontrast-enhanced computed tomography. **a** Multiple pulmonarymetastases in both lungs. **b** Consolidation suggesting inflammationapart from pulmonary metastasis. The arrow is pointing to the part of the tuberculosis infection

A 60-year-old Japanese man was diagnosed as having mesopharyngeal squamous cell carcinoma (T1N2bM1 – lung) at the Department of Head and Neck Surgery, Kyushu Hospital, in April 2017. He had been treated for pulmonary TB as an elementary school student, but on examination, thoracic CT and PET revealed no signs suggestive of inflammation, despite multiple pulmonary metastases (Fig. 1a). The first treatment attempted was a combination of cisplatin chemotherapy and local radiotherapy at 60 Gy (30 fr). The pulmonary metastases increased in size; therefore, weekly administration of paclitaxel + cetuximab combination therapy was initiated in August 2017 and a steroid (dexamethasone 10 mg) was simultaneously administered weekly. In October 2017, CT revealed consolidation, suggesting inflammation at loci other than the pulmonary metastases (Fig. 1b). Although our patient reported no subjective symptoms such as cough or fever, sputum was collected. A sputum smear test and real-time PCR yielded negative results, but *M. tuberculosis* was detected in a solid medium (“Ogawa” medium) culture test after 7 weeks. He was admitted to our hospital for TB treatment, and treatment of head and neck carcinoma was discontinued. He underwent treatment with anti-TB drugs, which were RFP, INH, EB, and pyrazinamide (PZA), but the cancer progressed and he died 2 months later. Because he had a history of TB infection, an IGRA test was not performed.

### Case 3

**Fig. 2:**
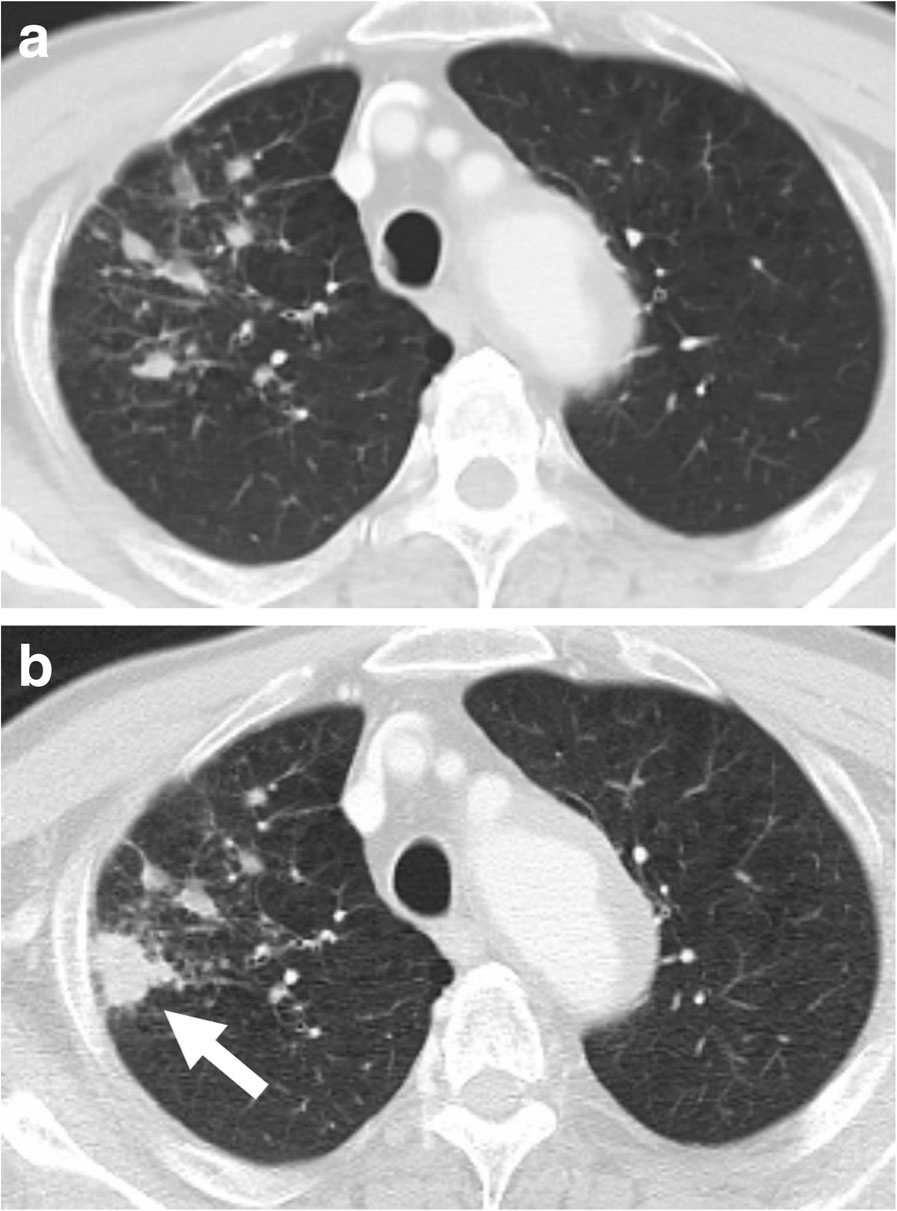
Chronological changes in the lungs for Case 2 as shown usingcontrast-enhanced computed tomography. **a** Multiple pulmonary metastases suspected in right lung. **b** Consolidation which appeared in a part of the pulmonary metastases. The arrow is pointing to the part of the tuberculosis infection

A 69-year-old Japanese man was diagnosed as having maxillary squamous cell carcinoma (T4aN0M1 – lung) at the Department of Head and Neck Surgery, Kyushu Hospital, in August 2016. He had no history of treatment for TB, and on examination, CT and PET revealed nodules, suspected to be pulmonary metastases, with mixed infiltrative opacity in the surrounding areas (Fig. 2a). He was treated with a combination of cisplatin chemotherapy and radiotherapy at 60 Gy (30 fr). However, locoregional control was not possible, and the pulmonary metastases increased in size; therefore, weekly paclitaxel + cetuximab combination therapy was initiated in May 2017 and a steroid (dexamethasone 10 mg) was simultaneously administered weekly. In November 2017, CT revealed partial expansion of the original consolidation (Fig. 2b). Although he reported no subjective symptoms such as cough or fever, sputum was collected. A sputum smear test and real-time PCR yielded negative results, but *M. tuberculosis* was detected in a solid medium (“Ogawa” medium) culture test after 4 weeks. Because TB was considered unlikely, an IGRA test was not performed. Treatment of head and neck carcinoma and treatment of TB was continued. He is currently alive 2 months after starting anti-TB drugs (RFP, INH, EB, and PZA).

## Discussion and conclusions

In 2000, the American Thoracic Society and the US Centers for Disease Control and Prevention presented the concept of latent TB infection (LTBI) [3]. LTBI refers to a condition in which the patient has *M. tuberculosis* infection that is indicated solely by a positive tuberculin reaction or a positive result in an IGRA test, with none of the following being observed: (i) clinical symptoms, (ii) bacteriological signs, and (iii) X-ray image findings suggesting TB. Vigorous treatment of LTBI is important for patients who are at high risk of developing TB. At present, this approach is an important part of the strategy for eradicating TB [2, 3].

Although TB is gradually decreasing in prevalence, the existence of patients in the high-risk group for TB presents new problems. The patients in this group are those with HIV infection or silicosis, those who have undergone organ transplantation, or those who are undergoing hemodialysis because of chronic renal failure [2, 3]. A group with an increased TB risk is also suggested to exist, in which the risk is increased but is less than that in the high-risk group for TB. This group also includes patients who are undergoing treatment with steroids or biological agents or have poorly controlled diabetes [2, 3]. In addition to this group, patients with malignant neoplasms are also at an increased risk of developing TB; the risk of this is highest with hematological malignancies, followed by head and neck carcinoma (Table 1) [3–5].

**Table 1:** Relative risk for developing active tuberculosis

Risk factor	Estimated risk of tuberculosis^a^	References
High-risk group		
1) HIV infection	35–170	[2, 3]
2) Chronic renal failure and/or hemodialysis	10–25	[2, 3]
3) Solid organ transplantation	20–74	[2, 3]
4) Silicosis	30	[2, 3]
Increased risk group		
1) Treatment with glucocorticosteroids	2–4	[2, 3]
2) TNF-α inhibitors	1.5–4	[2]
3) Diabetes mellitus	2–4	[2, 3]
Head and neck carcinoma	16	[4]
	2.86	[5]

*HIV* human immunodeficiency virus, *TNF* tumor necrosis factor

^a^Relative to persons with no known risk factors

However, there have been few reports on the onset of active TB during head and neck carcinoma treatment when active pulmonary TB was not observed at initiation of the treatment. There is no consensus on the approach for the treatment of head and neck carcinoma and/or TB in patients discharging *M. tuberculosis* [6, 7]. In practice, once a patient has developed TB, TB treatment is prioritized, although this does depend on bacterial discharge and infection severity. Therefore, the situation is one in which difficult choices have to be made, such as whether to suspend treatment of head and neck carcinoma, which may lead to progression of the cancer, or whether use of out-patient chemotherapy facilities must be restricted, due to the presence of other patients with cancer with low disease resistance.

Further, it is now possible to markedly extend the life expectancy of patients with head and neck carcinoma using novel maintenance drug therapies [8, 9]. This introduces the potential for an increase in the number of patients who readily develop TB due to regular steroid use and/or reduced immunity due to long-term maintenance chemotherapy.

Currently, patients with LTBI in the high-risk group for TB are prophylactically administered INH (an anti-TB agent) monotherapy. Although this treatment is strongly recommended, there are limited guidelines for patients with head and neck carcinoma [2, 3, 10, 11]. In the 1980s, the Memorial Sloan Kettering Cancer Center and MD Anderson Cancer Center stated that patients with head and neck carcinoma must be added to the guidelines, in addition to patients with a hematological tumor, as requiring prophylactic administration [10]. In addition, since then, there have been marked changes in the treatment of patients with cancer as well as in TB diagnostic methods. As a result, there are reports on the importance of reassessment of TB onset risks in patients with cancer and optimal management methods [11].

It is generally considered impractical and/or inefficient to conduct IGRA tests, sputum tests, and/or prophylactic administration of anti-TB agents in all patients with head and neck carcinoma. However, to avoid TB onset during cancer treatment, establishing a strategy for preventing TB onset before treatment is considered important from the viewpoints of the patient and the physician. Therefore, based on the experience with the three patients in this case series, it is suggested that it is efficient to investigate the need for prophylactic treatment, to initially focus on patients in whom TB onset would have a major impact on their social environment as well as the patients themselves, such as those who require long-term maintenance drug therapy. These are mainly patients treated in out-patient chemotherapy facilities where there are numerous patients with cancer with impaired disease resistance.

In addition, if lung field infiltrative opacity develops during treatment of head and neck carcinoma, particularly in asymptomatic patients, it is important to determine TB onset using TB tests with detection sensitivity before the spread of the contagious infection.

There have been no previous reports on active pulmonary TB onset during treatment of head and neck carcinoma; therefore, it is believed that this report will be useful for future studies.

## Abbreviations

CT: Computed tomography
EB: Ethambutol
HIV: Human immunodeficiency virus
IGRA: Interferon-γ release assay
INH: Isoniazid
LTBI: Latent tuberculosis infection
PCR: Polymerase chain reaction
PET: Positron emission tomography
PZA: Pyrazinamide
RFP: Rifampicin
TB: Tuberculosis
